# Amino acid racemization dating of marine shells: A mound of possibilities

**DOI:** 10.1016/j.quaint.2010.05.029

**Published:** 2011-07-01

**Authors:** Beatrice Demarchi, Matt G. Williams, Nicky Milner, Nicola Russell, Geoff Bailey, Kirsty Penkman

**Affiliations:** a BioArCh, Department of Chemistry, University of York, Heslington, York YO10 5DD, UK; b Department of Archaeology, University of York, King's Manor, York Y01 7EP, UK; c Scottish Universities Environmental Research Centre, Rankine Avenue, Scottish Enterprise Technology Park, East Kilbride G75 0QF, Scotland, UK

## Abstract

Shell middens are one of the most important and widespread indicators for human exploitation of marine resources and occupation of coastal environments. Establishing an accurate and reliable chronology for these deposits has fundamental implications for understanding the patterns of human evolution and dispersal. This paper explores the potential application of a new methodology of amino acid racemization (AAR) dating of shell middens and describes a simple protocol to test the suitability of different molluscan species. This protocol provides a preliminary test for the presence of an intracrystalline fraction of proteins (by bleaching experiments and subsequent heating at high temperature), checking the closed system behaviour of this fraction during diagenesis. Only species which pass both tests can be considered suitable for further studies to obtain reliable age information. This amino acid geochronological technique is also applied to midden deposits at two latitudinal extremes: Northern Scotland and the Southern Red Sea. Results obtained in this study indicate that the application of this new method of AAR dating of shells has the potential to aid the geochronological investigation of shell mounds in different areas of the world.

## 1 Introduction

Shell midden sites, found throughout the world, provide a range of important archaeological information, including the use of coastal resources, consumption practices and human impact on the environment. Shell middens are hard to define because they vary so greatly in size, content and form, but they are generally considered to be “a cultural deposit of which the principal component is shell” (Waselkov, 1987, p. 95). These deposits are especially found after the establishment of modern sea level in the mid-Holocene, and have been recorded in their hundreds of thousands around the coastlines of the world, often forming large mounds containing many millions of shells. Earlier deposits are much less frequent, most probably due to Holocene sea level rise, resulting in the submergence of palaeoshorelines and the associated archaeological evidence (Bailey and Flemming, 2008). However, earlier midden deposits are present in some areas of the world, often in deep cave sequences but also in some open-air locations. Middens from the Last Interglacial (Eemian) and before have been reported in Africa (e.g. Marean et al., 2007) and the Mediterranean (see Bailey and Milner, 2008), as well as on coastlines with steep offshore bathymetry or that have undergone tectonic uplift (e.g. O’Connor, 2007). The study of midden deposits, including their dating, must incorporate an accurate evaluation of the processes which have operated through time to produce the assemblages as they are observed today. Unfortunately, dating of these deposits can be problematic (e.g. Stein and Deo, 2003).

Radiocarbon dating is usually applied to develop chronological frameworks for shell midden deposits. It is a relatively costly procedure, so in most cases only a limited number of samples can be selected for dating; ideally these have to have an established provenance within a clearly identified stratigraphic sequence in order to produce reliable dating results for further interpretation. Shell middens, however, often do not meet these requirements. Often they accumulate relatively rapidly within the margins of error of radiocarbon dating, or are subject to high levels of disturbance, mixing and inversion of materials, requiring large sample sizes of dates to resolve issues of intra-site chronology (e.g. Glover et al., 1990; Stein and Deo, 2003).

Moreover, radiocarbon dating is complicated by factors such as the local spatial and temporal variation of the average global surface ocean marine reservoir effect (Stuiver and Braziunas, 1993; Ascough et al., 2005; Russell et al., in press). The source of the molluscan carbon, which partially depends on the feeding habits and metabolism of the molluscs (McConnaughey et al., 1997; Gillikin et al., 2005) and the possibility of mineral diagenesis (Taylor, 1987; Aitken, 1990) must also be considered.

In this context, a quick, cost-effective and (above all) reliable dating technique could be useful, allowing the processing of a large number of samples and an assessment of intra-site chronology and formation processes within shell midden deposits. This paper proposes intracrystalline protein geochronology on shell middens as a valuable “range finder” technique, providing qualitative relative age information, which could be calibrated by independent geochronology (such as radiocarbon). This could potentially be applied both to dating different layers within the same midden, when the temporal resolution is such that it is possible to resolve the internal stratigraphy, and also to correlate the age of different deposits on a regional scale (e.g. Bateman et al., 2008).

Amino acid racemization (AAR) dating of shell middens has a long history. As early as 1977, the Del Mar midden site (California) was targeted for AAR dating on *Chione* shells (Masters and Bada, 1977; Wehmiller, 1977). Masters and Bada (1977, 1978) compared the extent of isoleucine epimerization in radiocarbon dated shells and found distinct divergences. They attributed these to both inaccuracies in radiocarbon dating of carbonate and the isoleucine method on shell. Wehmiller (1977) showed that the shell radiocarbon and racemization data were consistent with shallow ground thermal effects. A third factor which was considered was the likelihood of sample mixing within the midden. Moreover, the possibility of burning and human-induced heating of edible molluscs is a general concern for AAR dating: exposure to high temperatures accelerates the degradation processes, resulting in high racemization values, which are not indicative of the age of a sample. If unidentified, this could significantly affect the reliability of the technique in archaeological contexts (e.g. Masters and Bada, 1978). Recent work includes the application of the conventional methods of AAR dating to shell mounds in South Africa and Northern Spain (Bateman et al., 2008; Ortiz et al., 2009).

The present work involves the application of recently introduced methodologies of AAR dating to shell material (Sykes et al., 1995; Kaufman and Manley, 1998; Penkman et al., 2008), which have been successfully used to date Quaternary terrestrial and marine sediments in the British Isles (Parfitt et al., 2005; Penkman et al., 2007; Davies et al., 2009). The main advance is in the isolation of a fraction of amino acids (intracrystalline) from the shell which behave as a closed system during diagenesis. The extent of protein degradation within this system can be used as a secure indicator of the age of a molluscan sample. The analysis of the intracrystalline fraction therefore represents an important step forward for the reliability of AAR dating of mollusc shells (e.g. Rose, 2009).

This paper shows how the recent advances in the AAR dating method can be effectively applied to shell midden deposits. The examples presented come from a range of samples from Holocene sites in Scotland (Latitude: around 55–57° N) and the Red Sea (Latitude: around 16° N). Detailed temporal and stratigraphical information was not available for all sites, hindering the possibility of considering shallow temperature burial effects. These can be particularly important for middens where the samples have not been submerged during burial and where the length of time at high (shallow) ground temperatures can be large in proportion to the age of the sample (Wehmiller, 1977). Within this study it was not possible to investigate the effect of different within-site thermal environments during burial: the aim was to compare the extent of racemization between archaeological deposits of significantly different age, and this pilot study generally considered one layer for each site.

The recent methodological advances in AAR dating are briefly summarized and a series of tests recommended to check for reliable AAR dating using the new closed system approach is proposed. A protocol is described which may be applied to obtain preliminary information on the suitability of different molluscan taxa for further archaeological/chronological investigation of previously unexplored areas. Finally, the reliability of the technique is tested on archaeological material associated with independent chronological information, and conclusions drawn on the utility of AAR dating for the dating of shell midden deposits.

The experiments investigate whether or not:

1. the taxa investigated (*Patella*, *Strombus*, *Tibia*, *Chicoreus*, *Trochus*, *Anadara*) retain an intracrystalline fraction of proteins;
2. the intracrystalline fraction behaves as a closed system with regard to protein diagenesis, both when artificially degraded via heating at high temperatures and in archaeological samples;
3. the new closed system approach of AAR dating can be applied to marine species from shell midden deposits;
4. AAR dating is able to discriminate between deposits of different ages within the Holocene;
5. exposure to high temperatures due to anthropogenic heating affects the degradation rates of the intracrystalline proteins.

## 2 Amino acid diagenesis in a closed system

### 2.1 Background

A mollusc shell contains both a mineral and a protein fraction; the biochemical functions of proteins in the process of biomineralization have been widely investigated, but many aspects still remain unclear (e.g. Marin et al., 2007). After the death of the organism, the proteins undergo diagenesis: they break down to smaller fragments by hydrolysis and the single amino acids racemize and decompose. Here, the term “protein” is used to indicate an original biomolecule (or a group of biomolecules) composed of a sequence of amino acids, which can be recovered from the biomineral upon death of the organism.

In some molluscan species, a fraction of the original proteins appears to be trapped within the mineral crystals and is thus protected from the external environment, forming a closed system (Penkman et al., 2008). Within this intracrystalline fraction, the extent of protein diagenesis is solely dependent on the thermal age of the fossil shells, i.e. the combined effect of time and temperature during burial upon the protein degradation reactions of the fossil shell. Conversely, the majority of the other proteins (intercrystalline) are not trapped in the crystals and therefore behave as an open system. The chemical isolation of the intracrystalline proteins is carried out by strong oxidation in sodium hypochlorite (bleaching) and complete removal of the intercrystalline component (Penkman et al., 2008; Demarchi, 2009).

The level of protein diagenesis is generally estimated by measuring the extent of amino acid racemization (AAR). Most of the amino acids can arrange their atoms in space in different configurations (called enantiomers) while maintaining their chemical properties: racemization is the reaction involving the transformation of one enantiomer into its non superimposable mirror image. When an amino acid differs from its mirror image, it is defined as a chiral amino acid. The majority of the natural amino acids possess at least one asymmetric carbon atom (a chiral centre), as a result of the four different substituents bonded to the alpha carbon. Amino acids with one chiral centre can exist in two non superimposable enantiomers, termed the l- (from the Latin *laevus*, laevorotatory or “left-handed”) and d- (from the Latin *dextro*, dextrorotatory or “right-handed”) forms. In living organisms, only l-amino acids are present, but they convert into their d-form after death; the ratio between the amount of d- and l- enantiomers (d/l value) will therefore indicate the time elapsed since death of a mollusc.

By analysing the extent of protein breakdown within the intracrystalline fraction, secure relative aminostratigraphies can be established for a series of molluscan samples: due to the progressive nature of diagenesis, the most degraded specimens are the oldest. This assumption is limited to: (i) closed system proteins; (ii) geographic areas which were exposed to the same climatic variations during the burial history of the fossil samples and (iii) monospecific samples, since different protein composition in different molluscan genera has an effect on the degradation (racemization) rates (the “species effect”) (e.g. Lajoie et al., 1980).

### 2.2 Chiral amino acid analysis

Chiral amino acids from the intracrystalline fraction were detected following the method by Penkman et al. (2008). Here, the main steps used for sample preparation and the chromatographic analysis of multiple amino acids, performed with a modified method of Reverse Phase High Pressure Liquid Chromatography (RP-HPLC) of Kaufman and Manley (1998), are briefly reported.

Each shell was sub-sampled for amino acid analysis, by snapping off a fragment of a few square millimeters; for *Patella*, the edge of the shell was specifically targeted, to provide a consistent calcitic structural layer for analysis (Demarchi, 2009). Each shell fragment was first sonicated and rinsed at least five times in ultrapure water (18.0 mΩ), then air-dried and crushed with a quartz mortar and pestle. Powdered samples were weighed out (1–10 mg) and 50 μL 12% NaOCl (BDH) per mg of powdered shell was added at room temperature. The powders were left to soak for 48 h, and vortexed after 24 h to ensure complete penetration of the bleaching agent. The 48 h bleaching step is effective for isolating the intracrystalline proteins in a number of molluscan species (Penkman et al., 2008; Demarchi, 2009).

After the bleaching agent was removed by washing in ultrapure water (5 cycles) and methanol (1 cycle), the dry powders were further split into two subsamples. This allows the analysis for each sample of both the free amino acid fraction (“FAA”), representing the amino acids which are not protein-bound, and the total hydrolysable amino acids (“THAA”), representing both the bound and unbound amino acids.

For the analysis of the FAA, the subsamples were accurately weighed into sterile 2 mL glass vials and demineralised in 10 μL/mg cold 2 M hydrochloric acid (HCl) to dissolve the carbonate. Protein-bound amino acids were released by adding 20 μL per mg of sample of 7 M HCl to the bleached powder and hydrolysing at 110 °C for 24 h. All samples were dried in a centrifugal evaporator and rehydrated with 10 μL per mg of rehydration fluid, enabling quantitative analysis of the amino acids via RP-HPLC.

For analysis, a modified analytical method of Kaufman and Manley (1998) for an automated system of RP-HPLC, described in Penkman (2005), was adopted, allowing the routine analysis of l and d isomers of 10 amino acids. A number of advantages result from the use of this modified RP-HPLC method. Firstly, the size of sample required is very small, <2 mg. This can be crucial for the analysis of archaeological material, which is often scarce or too precious to analyse destructively using large samples. Secondly, the high automation of the system allows for maximum efficiency of the analysis, increasing the number of samples which can be processed, minimising the analytical variability and therefore improving the statistical significance of a given dataset. Moreover, it is possible to detect the enantiomers of multiple amino acids, thereby increasing the level of resolution available. The conventional method of AAR dating generally focused on the racemization (epimerization) of a single amino acid, isoleucine (e.g. Miller et al., 1999). It has been reported that the d/l values of different amino acids exhibit a very strong co-variance (Goodfriend, 1991; Kaufman and Manley, 1998). It follows that unexpected differences in the dl ratios of some amino acids can be used to detect compromised samples (see Section 4.3). The technique proposed is therefore cost-effective and efficient, and yields accurate quantification of multiple amino acids in the sub-picomole range. It is expected that the concentration of amino acids in the intracrystalline fraction would be lower than in the whole shell. Therefore this method is highly appropriate for testing the ability of the bleaching treatment to isolate the amino acids from the intracrystalline fraction.

## 3 Isolation and testing of a closed system of amino acids in marine shells

### 3.1 Bleaching and leaching tests: rationale

Not all molluscs are suitable for closed system AAR dating and tests must be performed to investigate the behaviour of protein degradation in each taxon. This paper presents results on six different taxa: *Patella*, *Strombus*, *Anadara*, *Tibia*, *Chicoreus*, *Trochus*.

Bleaching tests are done in order to optimise the efficiency of bleaching in isolating the intracrystalline amino acids (Penkman et al., 2008). Extensive bleaching and heating experiments on modern specimens were performed for *Patella* and a large database collected (Demarchi, 2009). Bleaching tests demonstrated that *Patella* retains a fraction of intracrystalline proteins which can be isolated by a 48 h bleaching step. The concentration of amino acids in bleached shells (∼5 nmol/mg) represents about 13% of the original (unbleached) concentration (∼38 nmol/mg) (Demarchi, 2009). The amino acid (THAA) concentrations are relatively high even after the matrix proteins are removed, allowing precise quantification via RP-HPLC without the interference of the background noise (generally, around 2–10 pmol/mg). The effectiveness of 48 h bleaching in isolating the intracrystalline amino acids from *Patella* is in agreement with the data from other shell taxa analysed in the NEaar laboratory. A 48 h bleaching step was therefore adopted for *Strombus*, *Anadara*, *Tibia*, *Chicoreus* and *Trochus*.

The isolation of a fraction of intracrystalline proteins is critical for the use of molluscan material for reliable AAR dating, as a mollusc shell does not represent a closed system *per se* (Brooks et al., 1990). However, the behaviour of this fraction during long term diagenesis could be such that it is inappropriate to use it for dating purposes. High-temperature (kinetic) experiments have traditionally been performed to monitor the behaviour of protein diagenesis, particularly amino acid racemization (AAR), within laboratory timescales (e.g. Hare and Mitterer, 1969). Heating experiments can be performed to test the closed system behaviour of the intracrystalline proteins from any molluscan species, and check whether leaching of the amino acids from the biomineral into the external environment occurs. The best strategy to enable quantification of the loss of amino acids from the system is to perform the heating experiments in an excess of water (Collins and Riley, 2000; Penkman et al., 2008): if the amino acids were contained within a closed system, no amino acids should be detected in the water used for the heating experiments.

**Fig. 1:**
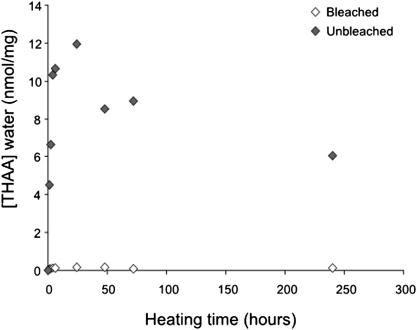
Leaching of THAA amino acids into water from unbleached (whole shell) and bleached modern *Patella* shells upon isothermal heating (*T* = 140 °C). Note that unbleached shells lost up to 12 nmol/mg of amino acids into the water within the first 24 h of heating. On the contrary, the loss of amino acids from bleached shell powder is negligible.

A number of tests were performed to investigate the reliability of *Patella* for protein geochronology (Demarchi, 2009). These involved heating bleached and unbleached shell powder at 140 °C and for different times (between 1 and 240 h). The amino acid concentrations were measured for both the powder and the water to quantify leaching of amino acids from bleached/unbleached powders. For the bleached shells the concentration of amino acids in the water was similar to background levels. In contrast, the concentration of amino acids in the water for unbleached shells was three orders of magnitude higher (nanomoles) (Fig. 1). This demonstrates that leaching is particularly marked for whole-shell (unbleached) *Patella*, which therefore does not represent a closed system. The implication is that the d/l values measured in unbleached shells would not necessarily be representative of the age of the mollusc, since such a permeable system is particularly prone to external contamination and to be affected by environmental factors (e.g. pH of the burial soil). On the contrary, the intracrystalline amino acids in *Patella* approximate a closed system with regard to diagenesis, thus providing a robust substrate for reliable AAR dating (Demarchi, 2009).

### 3.2 A quick exploratory test for closed system behaviour of new species

Bleaching and heating experiments on modern samples are crucial for assessing the reliability of molluscan taxa for the closed system approach of AAR geochronology. However, such rigorous testing of each species is time consuming. This is a disadvantage when pilot data are required to assess the suitability (or otherwise) of different shell taxa which have never been previously investigated for closed system AAR. This information is useful during archaeological excavations in order to optimise the sampling strategies on-site and to develop research plans which include a detailed AAR investigation of the deposits.

The first application of closed system AAR dating to shell middens from a tropical area (Farasan Island, Southern Red Sea), is described below. In order to test the suitability of the species available from the Red Sea middens, a simple initial experimental test for closed system behaviour was devised, which can be performed directly on archaeological shells from a site. It is recommended that these tests should be performed routinely on new species of shells in order to provide an initial assessment of the potential of protein geochronology for each different taxon and site.

Five species of shells, from different midden sites and among the most abundant in the archaeological record in this area, were targeted and the results obtained were used for informing further field sampling (see Section 4.5): *Chicoreus* sp., *Tibia insulaechorab curta*, *Strombus fasciatus*, *Trochus dentatus*, *Anadara erythraeonensis.* These shells were collected from a group of middens located in an area north of the Harid bay, and are likely to be broadly contemporaneous based on their inland location and linear distribution (Williams, in preparation). *Chicoreus* shells were collected from a basal layer in the shell midden.

The bleached shells were heated at high temperature in sealed glass tubes under hydrous conditions to test for closed system behaviour. For each sample, ∼20 mg of dry bleached powders were weighed into sterile glass ampoules and 300 μL of ultrapure water was added. The glass ampoules were sealed and placed in an oven at 140 °C for two different times (24 and 48 h), with a “zero” time-point (unheated). This was performed for each of the five taxa. Three replicates were prepared for each time-point.

After heating, 100 μL of the supernatant water was removed and analysed for the free amino acids (“FAAw” fraction; dried in a centrifugal evaporator and rehydrated) and 100 μL was removed and analysed for the total hydrolysable amino acids (“THAAw” fraction; hydrolysed using 6 M HCl as no demineralisation of shell is needed, 20 μL per mg/equivalent). The heated powder was air-dried and separated into two sub-samples, for the analysis of the “FAA” and “THAA” fractions, performed following the analytical method detailed in Section 2.

The main aim of the heating experiments was to test whether:

• a fraction of intracrystalline proteins exists within each taxon of shells and can be isolated via bleaching;
• the intracrystalline proteins behave as a closed system during diagenesis, by mimicking natural degradation with exposure to high temperatures. If this is the case, no leaching should occur; it is also expected that with increasing heating time, the extent of racemization (d/l) will increase, as well as the percentage of amino acids produced by hydrolysis of the peptide chain (free amino acids, FAA) and the extent of decomposition of certain amino acids (e.g. serine into alanine) (Penkman et al., 2008).

### 3.3 Existence of the intracrystalline fraction

**Fig. 2:**
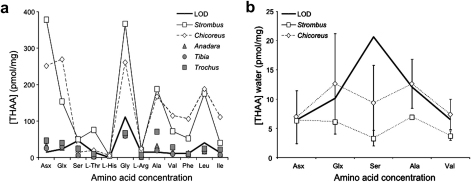
(a) THAA concentration (powder) for unheated intracrystalline *Strombus*, *Chicoreus*, *Anadara*, *Tibia* and *Trochus* samples. Note that only *Strombus* and *Chicoreus* intracrystalline values can be considered significantly higher than the limit of detection. The Limit Of Detection (LOD) was calculated on the basis of the amino acid concentration detected in procedural blanks used in this study: LOD = *X*_blank_ + *k*^∗^*σ*_blank_; where *X*_blank_ is the mean of the blank measures, *σ*_blank_ is the standard deviation of the blank measures, and *k* = 3. (b) Loss of amino acids in water for *Strombus* and *Chicoreus* after 48 h of isothermal heating at 140 °C, compared to the LOD.

*T. insulaechorab curta*, *T. dentatus* and *A. erythraeonensis* showed very low concentrations of intracrystalline amino acids in the bleached powders, in both the FAA and the THAA fractions. The concentrations were comparable with background levels and, in most cases, fell below the limit of detection (Fig. 2a).

The low protein contents detected may be due to the sampling strategy, as each shell was sampled only in a single location. It is therefore possible that this specific sampling area was enriched in mineral, but very poor in proteins, leading to the low amino acid concentrations observed. Further investigation of possible sampling strategies may help clarify this issue. The data recovered from *Anadara*, *Trochus* and *Tibia* were therefore not meaningful for the interpretation of intracrystalline protein diagenesis patterns. The results concerning the intracrystalline fraction within *Anadara* are particularly interesting, as this genus has been used in the past for traditional whole-shell AAR geochronology (e.g. Kimber and Griffin, 1987; Murray-Wallace et al., 1991). On the contrary, both *Strombus* and *Chicoreus* samples showed high concentrations of amino acids in the intracrystalline fraction and so were tested further (Fig. 2a).

### 3.4 Testing the closed system in *Chicoreus* and *Strombus*

The intracrystalline fraction isolated from *Chicoreus* and *Strombus* was tested for closed system behaviour via leaching experiments; for both species, the concentration of free and total hydrolysable amino acids detected in the water was only ∼10 pmol/mg higher than background levels after 48 h of heating at 140 °C (Fig. 2b). No significant amounts of amino acids were leached out of the intracrystalline fraction, which therefore appears to behave as a closed system.

**Fig. 3:**
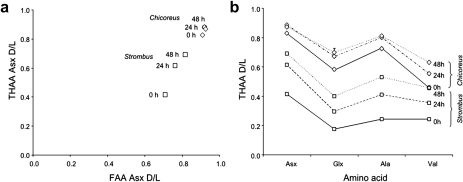
(a) Asx THAA vs FAA dl ratio for *Strombus* and *Chicoreus*. Note the increase in d/l values for *Strombus* with increasing heating time, while *Chicoreus* Asx d/ls cluster at higher values. Error bars represent one standard deviation around the mean. (b) “Spider diagram” illustrating the increase of THAA d/l values for Asx, Glx, Ala and Val with increasing heating time, for *Strombus* (square symbols) and *Chicoreus* (diamond symbols). Error bars represent one standard deviation around the mean.

Further confirmation of the closed system behaviour of the intracrystalline proteins was sought by examining the diagenesis patterns by means of THAA vs FAA d/l plots (Fig. 3a) and “spider diagrams” (Fig. 3b) (e.g. O’Neal et al., 2000; Wehmiller et al., 2009). In a closed system, the extent of racemization measured in the FAA and the THAA fractions should be highly correlated. When the THAA d/l values are plotted against the FAA d/l values (Fig. 3a):

• a series of samples should fall on the same trendline;
• the less degraded samples should plot near to the origin of the axes, with progressively more degraded samples plotting towards the top right corner of the graph.

A sample falling outside the expected trajectory is likely to have been compromised (e.g. Preece and Penkman, 2005).

Spider diagrams are useful for testing the consistent behaviour of multiple amino acids within the system (Wehmiller et al., 2009): lines connecting d/l values measured on the same species should not cross and the d/l values should increase with increasing heating time. This is the case for the amino acids which yielded the best chromatographic resolution and were therefore targeted for this study: aspartic acid/asparagine (Asx), glutamic acid/glutamine (Glx), alanine (Ala) and valine (Val) (Fig. 3b). Serine (Ser) follows unusual racemization patterns, with d/l values increasing very fast with increasing heating times, but then decreasing after a maximum, thus displaying an upward convex trend (Penkman et al., 2008; Demarchi, 2009). Therefore Ser was not included in the spider diagram in Fig. 3b. The racemization values for the species analysed were very high, as expected for an area of low latitude such as the Farasan Islands, where the higher temperatures would accelerate the reaction.

It is expected that Asx would enable good levels of resolution for Holocene sites, due to its high racemization rates (Goodfriend, 1992; Collins et al., 1999; Barbour Wood et al., 2006; Hearty and Kaufman, 2009). However, the high temperature experienced by Red Sea samples during their burial history limits the use of Asx as an age indicator at these latitudes. In unheated *Chicoreus* samples the Asx d/ls were around 0.9, indicating that the reaction is approaching equilibrium. However, for *Strombus* the extent of racemization was lower (Fig. 3a and b), and a clear increase in both THAA and FAA d/l values was observed with increasing heating time. This could potentially be due to age differences between samples/midden sites (*Chicoreus* shells came from a basal layer within one of the middens), as well as taxon-specific protein degradation patterns. A tight relationship between the FAA and the THAA d/l values of the other amino acids could also be seen for *Chicoreus* and *Strombus*, thus confirming the closed system behaviour of the intracrystalline proteins from these two species (Supplementary information).

**Fig. 4:**
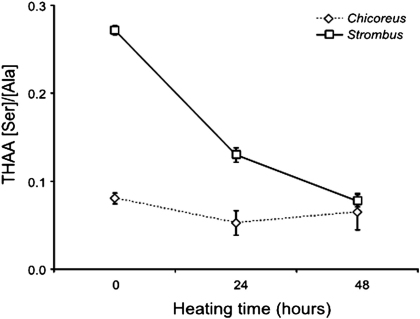
Decrease of the THAA [Ser]/[Ala] value with increasing heating time, for *Strombus* and *Chicoreus*. This represents the decomposition of Ser into Ala with the progression of diagenesis. Note that the THAA [Ser]/[Ala] value for unheated *Chicoreus* is close to zero, thus indicating that this shell already displays a high extent of protein diagenesis. Error bars represent one standard deviation around the mean.

Two more protein breakdown indicators can also be considered when testing the behaviour of a molluscan species with regard to protein diagenesis: the percentage of free amino acids and the ratio between the concentrations of serine and alanine (Bada et al., 1978; Penkman et al., 2008). Both species analysed showed increasing percentages of free amino acids with increasing heating time, with *Chicoreus* displaying more extensive degradation (Supplementary information). A net decrease in the [Ser]/[Ala] value could be observed for *Strombus* values, while *Chicoreus* seemed to have a slight (but not statistically significant; two-tailed *t*-test, SigmaPlot, v.11, *p* = 0.552) increase at higher heating times (Fig. 4). This is likely to be due to the decrease in analytical precision at the low concentrations of Ser (similar to background levels, see Supplementary information) at these higher levels of protein decomposition, due to the quasi-exponential nature of diagenesis.

### 3.5 Conclusions on heating tests for the Red Sea

Bleaching and heating tests can be used as a quick, routine protocol to detect the most suitable shell substrate to be targeted for geochronological investigations of a new geographical and climatic area. Three out of the five taxa targeted for the Red Sea pilot study show very low protein content in the intracrystalline fraction: *T. insulaechorab curta*, *T. dentatus* and *A. erythraeonensis.* As the amino acid concentration for these species is barely distinguishable from the background noise, the collection of these shells from archaeological sites for intracrystalline AAR studies is not recommended at this preliminary stage. However, further research may be able to clarify if this is a bias introduced by sampling in areas of the shell characterised by low proteic content.

The data recovered from *Strombus* and *Chicoreus* samples show significantly higher amino acid concentrations, closed system behaviour and a general pattern of increase in protein degradation (increase in d/l values, increase in percentage of FAA, decrease in [Ser]/[Ala]) with increasing heating time. They are therefore suitable for targeting for intracrystalline AAR dating. This initial assessment protocol thus provides useful information to aid the sampling strategies of excavations (Section 4.5).

## 4 Archaeological tests

The bleaching and heating tests demonstrated that *Patella*, *Strombus* and *Chicoreus* retain an intracrystalline fraction of amino acids which behave as a closed system during diagenesis. These taxa were therefore used for the AAR investigation of shell-bearing archaeological deposits from two geographic areas: Scotland (UK) and the Farasan Islands (Saudi Arabia). The extent of protein degradation was compared with available independent age information. Because of the persistence of a species effect within the closed system as well as the difference in thermal regimes between Scotland and the Red Sea, the extent of protein degradation cannot be directly compared.

### 4.1 Patella from Scottish sites

This section describes the results obtained on fossil *Patella* shells from different archaeological sites in Scotland. *Patella* specimens were analysed from two shell midden sites (Table 1): Sand (Mesolithic) and Coire Sgamhadail 1 (Neolithic/Bronze Age) (Hardy and Wickham-Jones, 2009). Independent chronometric information was available, enabling the testing of the ability of closed system AAR to distinguish samples of different ages.

Sand and Coire Sgamhadail 1 are two of the sites targeted in the *Scotland’s First Settlers* project, a regional archaeological investigation of the Inner Sound, Wester Ross (Hardy and Wickham-Jones, 2009). Sand is a rock shelter associated with a well-preserved shell midden which shows evidence for discontinuous human occupation from as early as the late 7th millennium BC to the Neolithic. Samples analysed for AAR dating (courtesy of P. Ascough, K. Hardy and C. Wickham-Jones) come from layer B24A NE spit 5. This layer is bracketed by two radiocarbon dates (Ascough et al., 2007; Hardy and Wickham-Jones, 2009):

• 7050–6500 cal BC (OxA-10384): obtained from ^14^C on a bevel-ended bone artefact BT03 from Spit 4, retrieved from a loose unconsolidated limpet midden overlying a rockfall and covered by crushed shell and turf;
• 7050–6450 cal BC (OxA-12096): obtained from ^14^C on a second bevel-ended bone artefact, BT30, from sample B25B NE Spit 7 collected from the same limpet midden.

The overall age span for the AAR samples is therefore 7050–6450 cal BC.

Coire Sgamhadail 1 has a small assemblage of shells and cultural material. Two radiocarbon determinations have been reported (Hardy and Wickham-Jones, 2009):

• the first obtained from a piece of hazel charcoal found in a cave shell midden, Test Pit 1, context 8914 (2550–1950 cal BC, AA-50692)
• the second from an ungulate bone securely stratified in the same shell midden, Test Pit 1, context 8914 (2290–1880 cal BC, AA-50693)

For the purpose of this study, it was therefore assumed that the site age ranged between 2550 and 1880 cal BC.

Two sites of historic age were also considered. Although not shell midden sites, as defined above, they do contain samples of the same taxon that allows the technique to be tested on younger material.

Samples NEaar 5419–5422 (Table 1) come from Archerfield, a recently discovered “lost” Medieval village in the East Lothian. Currently unpublished, it represents one of the rare rural Medieval settlements found in Scotland. *Patella* specimens from Archerfield were dated directly at the East Kilbride radiocarbon facility alongside contemporaneous terrestrial carbonised plant material for investigations regarding the marine reservoir effect (MRE). The terrestrial samples (SUERC19680–81, SUERC19685–90) yielded a mean date of 490 ± 25 BP, corresponding to a calibrated date range of 1410–1445 AD at 95.4% confidence (atmospheric data from Reimer et al., 2004; Oxcal v 3.10 Bronk Ramsey, 2005; Russell et al., in press).

Whitegate Broch in Caithness was excavated as part of The Caithness Broch Landscapes Project in 2002, with the first stage focusing on the re-survey and excavation of broch settlements (circular hollow-walled buildings, commonly found in Scotland) examined by Sir Francis Tress Barry in the second half of the nineteenth century (Anderson, 1901). Excavations in 2006 provided sample material including shell and animal bone from the interior of the broch structure (Heald and Jackson, 2001). *Patella* shells were radiocarbon dated for MRE investigations at SUERC alongside contemporary terrestrial herbivore bones. The bone material provided a mean date of 1370 ± 70 BP, which calibrated to a calendar age range of 880–1210 AD at 95.4% confidence (Russell, unpub. data) using Oxcal v. 3.10.

### 4.2 Racemization results for the Scottish sites

For shells which have undergone diagenesis in the burial environment, the extent of racemization within a closed system of proteins should be directly related to the age of the fossil sample (Penkman et al., 2008).

**Fig. 5:**
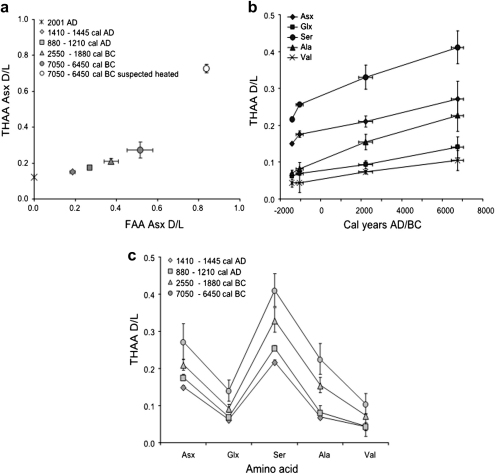
(a) FAA vs THAA plot for Asx d/l measured in Scottish archaeological *Patella*; error bars represent one standard deviation around the mean for each site. Modern (collected in 2001 AD) *Patella*d/l values are also plotted for comparison. (b) Asx, Glx, Ser, Ala and Val THAA d/l values measured in Scottish archaeological *Patella* plotted against the age of each site. Error bars represent age uncertainty on the *x*-axis and one standard deviation around the mean of d/l values for each site on the *y*-axis. (c) “Spider diagram” illustrating the increase of THAA d/l values for Asx, Glx, Ser, Ala and Val with increasing age of the archaeological deposit. Note that different amino acids have different resolving powers over different timescales. Error bars represent one standard deviation around the mean.

Archaeological *Patella* samples from the Scottish sites detailed in section 4.1 were analysed and the dl ratios of multiple amino acids represented on FAA vs THAA plots, as described for the heating experiments on *Strombus* and *Chicoreus* (Fig. 3a). All values fell on a trendline for Asx (Fig. 5a) as well as Ser, Ala, Glx, and Val (data not shown), thus satisfying the first condition for closed system behaviour. All the amino acids considered displayed the expected increase in dl ratios with increasing age when THAA and FAA d/l values for each subsample were compared to independent geochronological information (Table 1, Fig. 5a–c). However, not all the amino acids allow the same resolution over different timescales. Different amino acids racemize at different rates as a function of their molecular structure, position in the protein chain, flanking residues and status (free or bound; e.g. Kriausakul and Mitterer, 1980). Ser and Asx are among the fastest racemizers and will therefore give better resolution over Holocene timescales (Fig. 5b and c). The fast racemization rates of Asx have been successfully used to date young samples in both warm (Goodfriend, 1992; Barbour Wood et al., 2006; Hearty and Kaufman, 2009) and cold (e.g. Goodfriend et al., 1996) climates. Data obtained in this study confirm the possibility of applying the technique to extremely young samples, even at the low burial temperatures experienced by the shells in Scotland.

Samples from Archerfield and Whitegate could be distinguished on the basis of Ser and Asx THAA and FAA dl ratios (Fig. 5a–c). The Mesolithic and Neolithic/Bronze Age shell middens of Sand and Coire Sgamhadail 1 can also be separated on the basis of the d/l values measured in *Patella* (Fig. 5b and c), as well as the decomposition rate of Ser into Ala (Supplementary information). However, the variability of the data was higher in these contexts. This is unsurprising and it is likely to be due to the extremely complex depositional patterns of midden deposits. Mixing can affect the sample’s deposition even at small scales, causing adjacent shells to yield different d/l values. Also, the effect of variable thermal environments must be considered, as shell midden samples may have been exposed to solar radiation and natural heating during shallow ground burial (Wehmiller, 1977). Here only a single layer for each site was considered, and therefore it was not possible to assess the effect of shallow burial thermal environments in detail. However, a comprehensive study of a single shell midden in Skye, Scotland, will provide the data to investigate these issues further. Other studies (e.g. Barbour Wood et al., 2006) have reported better age resolution for other biominerals using the racemization of aspartic acid than that observed for these Scottish shell middens. However, these were undertaken in significantly warmer latitudes: the colder burial temperatures experienced by the Scottish *Patella* shells decelerate the racemization rate significantly, with a consequent loss in resolution.

### 4.3 Anthropogenic heating detected?

One problem that may affect AAR dating of edible shells is the common practice of food processing, which may involve heating (Masters and Bada, 1977, 1978), thereby inducing protein degradation unrelated to the age of the sample. Detecting whether a shell has been exposed to very high temperatures is of the utmost importance, as not doing so may result in misleading conclusions on the age range of a sample. If only a few samples are analysed from a midden site where the likelihood of anthropogenic heating is high, and no comparative concentration data are available for the shell taxon analysed, the dl ratios alone may result in an overestimate of the age of the heated specimens.

Having identified heating (burning in campfires or brush fires) as being the probable source of anomalous d/l values in archaeological ostrich eggshells, Brooks et al. (1991) performed a series of heating experiments and described the resulting sequence of changes in amino acid composition and concentrations. In particular, 1 hour of dry heating of ostrich eggshell at 200–280 °C caused the amounts of aspartic acid, glycine and alanine to decrease with respect to the unheated composition. Serine, threonine and arginine were either only detectable at trace levels or had disappeared completely, while the concentration of glutamic acid remained relatively constant (Brooks et al., 1991).

**Fig. 6:**
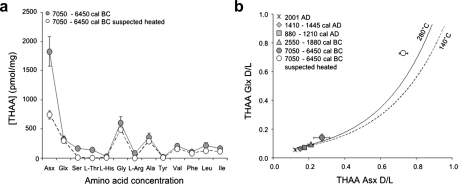
(a) THAA amino acid composition for unheated and “suspected heated” Sand samples. Error bars represent one standard deviation around the mean. (b) THAA Glx vs THAA Asx d/l plot for unheated and “suspected heated” Sand samples. The trendlines for heating experiments at 140 °C and 280 °C, performed on modern *Patella* (Demarchi, 2009), is also reported: note that the “suspected heated” samples from Sand fall on a different trajectory, possibly indicating different diagenetic pathways induced by exposure to very high temperatures due to fire.

Anomalous d/l values were found for two of the samples from Sand, which differ from other values from the same site. These did not appear macroscopically “burnt” or charred. However, when the compositional data are considered (Fig. 6a), a similar pattern to that described by Brooks and colleagues is shown, especially regarding the decrease in aspartic acid, serine and arginine. Currently, heating experiments at 280 °C on bleached *Patella* are in progress. Preliminary results appear to confirm the same trend as observed on ostrich eggshell. On the contrary, other *Patella* samples analysed in the NEaar laboratory, from a ∼250 ka raised beach deposit in Northern England, show a “normal” concentration profile, different from the “suspected heated” samples (Demarchi, 2009). This indirectly confirms that the d/l values of two of the Sand samples had been artificially raised by heating and do not represent real age differences. The chromatograms for “suspected heated” samples also show a striking difference when compared to “normal” samples, displaying not only the relative compositional differences described above, but also the presence of a number of other peaks that do not correspond to known standards. These peaks are postulated to be peptide/amino acid degradation products and could potentially be identified using mass spectrometry.

Moreover, samples suspected of exposure to very high temperatures could be easily recognised on a plot of THAA Glx d/l vs THAA Asx d/l (Fig. 6b). Glx and Asx d/l values should covary very strongly; a plot of Glx vs Asx d/l is able to identify aberrant results and can therefore be used as a criterion for screening results in AAR studies (Kaufman, 2006). The Sand “suspected heated” samples fall off the trajectory of both archaeological and modern *Patella* heated at 140 °C (Demarchi, 2009). However, the preliminary data for the heating experiments at 280 °C fall on a trajectory which approaches more closely that of the Sand samples (Fig. 6b). This is believed to be due to the fact that different diagenetic pathways are followed by the proteins at the relatively low temperatures of the kinetic experiments at 140 °C compared to the significantly higher temperatures when exposed to fire. These can easily reach 500 °C and above, although such temperatures have been observed to induce charring of bone (Brain and Sillen, 1988). The data from the *Patella* heated at 280 °C show a trend towards that of the “suspected heated” Sand sample, supporting the hypothesis. The identification of anthropogenic heating is especially important for shells not appearing macroscopically “burnt” or charred, since it provides evidence on the use of fire on a site.

### 4.4 Conclusions for Scottish shell middens

Overall, given that suspected heating appears to be identifiable, amino acid data from *Patella* shells provide a relatively simple method for distinguishing Holocene deposits on the basis of their mean d/l ratios. The best amino acids to target for dating sites younger than 12 ka are the fast racemizing amino acids such as Ser and Asx: the d/l values measured over a range of Holocene shells were able to correctly discriminate these deposits of different ages. The resolution obtained was surprisingly good for the historic samples of Whitegate and Archerfield, which differ in age by only a few hundred years. The two midden sites of Coire and Sand could also be distinguished on the basis of the extent of racemization as well as the [Ser]/[Ala] values. The resolving power of the technique is less for these older sites, mainly due to the higher natural variability of the data. This is likely to be due to sample mixing within the shell midden and shallow ground burial temperature differences. However, the two sets of data fall in two definite clusters on a THAA vs FAA plot, and can therefore be distinguished.

This has important implications for the archaeological application of AAR dating to shell midden deposits in Scotland, where the high concentration of mounds implies that radiocarbon can not be used feasibly as a “range finder” technique. This study has demonstrated that AAR can be used as a “screening” method, to build relative chronological frameworks and group the deposits in broad age ranges. Radiocarbon can then provide numerical age information to calibrate the relative framework.

### 4.5 Testing the Red Sea shell middens

Shell middens were first systematically recorded in the Farasan Islands, southern Red Sea, only in 2006 (Bailey et al., 2007). Over 1500 shell midden sites have been found on the main islands of the Farasan archipelago, distributed in ten large concentrations (Williams, in preparation). The islands are c. 40 km from the Saudi mainland, implying that those responsible for the formation of the midden sites must have been competent in sea travel. Little archaeological work has been undertaken on the islands, and the origin and extent of these deposits remains unclear. Preservation of the sites is excellent, owing to a combination of environmental factors and the current low population density on the islands.

*S. fasciatus* proved to be present in most shell mounds across the islands, and it was selected for further testing to determine the extent to which the temporal resolution of sites could be assessed. Given the scale of the preserved deposits, excavation focused on two sites on the main island, both of which are at risk due to development activities.

Independent radiocarbon age information was obtained for these two sites and AAR analyses undertaken on the intracrystalline proteins from *S. fasciatus*. AAR samples from KM1057 and JE0004 were taken directly from sections exposed during excavation, assuring their stratigraphic integrity, and adjacent to the respective radiocarbon dating samples (3641–3372 cal BC and 3519–3126 cal BC, respectively: Table 2). A third sample for protein geochronology was taken from the base of a geoarchaeological trench (designated KM1367), excavated in the centre of the in-filled Khur Maadi bay, which had yielded younger radiocarbon ages (1520–1270 cal BC: Table 2). Unfortunately, radiocarbon determinations were not performed on the same samples used for AAR dating. Therefore, the possibility that the radiocarbon ages may not be representative of the age of the shells targeted for amino acid analysis can not be excluded. However, for the purposes of this study it was assumed that little or no sample mixing had occurred within the layers.

Ten *S. fasciatus* shells were sampled from each context and analysed according to the bleaching protocol described in Section 2. These were used to test the resolution of the method, and assess the extent to which AAR could be used to distinguish between deposits of different age in this area.

**Fig. 7:**
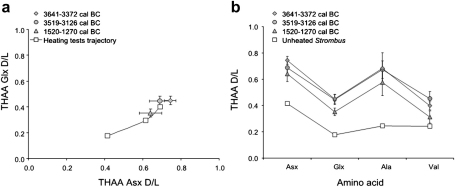
(a) Glx vs Asx THAA d/l plot for *Strombus* samples investigated for the closed system test (white symbols) and for the archaeological applications on the Farasan island shell middens. Note that all samples fall on a coherent trajectory of protein degradation. Error bars represent one standard deviation around the mean. (b) “Spider diagram” for archaeological and “test” unheated *Strombus* shells from the Farasan Islands comparing the THAA d/l values for Asx, Glx, Ala and Val with the calibrated ^14^C ages of the archaeological layers.

A plot of THAA Glx d/l vs THAA Asx d/l showed that all the samples fall on a coherent trajectory of increase in the extent of degradation, thus confirming the good closed system behaviour of the intracrystalline fraction. Samples from JE0004, KM1057 and KM1367 displayed a higher extent of protein degradation than the unheated “test” *Strombus* samples (Fig. 7a). This was also confirmed when Asx, Glx, Ala and Val THAA d/l values were considered (Fig. 7b). This provides a maximum age for these specimens, indicating that the test samples are younger than 1520–1270 cal BC.

Data from JE0004 and KM1057 are indistinguishable within one standard deviation around the mean for each site. This is consistent with the radiocarbon ages, and it suggests near-contemporaneous deposition of the two layers. KM1367 showed lower levels of protein degradation for Asx, Glx, Ala and Val (Fig. 7a and b), indicating that this layer is likely to be younger, again in agreement with the radiocarbon dates. However, the difference is very small; Glx and Val are the only amino acids for which this difference is not within one standard deviation. Glx and Val racemization rates are generally lower than for Asx and Ala, therefore these amino acids have good potential for enabling age resolution of samples at these latitudes and within this time interval.

### 4.6 Conclusions on the Red Sea

Both heating tests and the comparison with numerical radiocarbon dates showed that the new methodologies of AAR geochronology have the potential to be applied to the dating of shell midden deposits in tropical areas, where high temperatures prevail, such as the Farasan Islands. Further analyses are being undertaken to determine the extent to which this method can be used to distinguish between sites of different age at a broader temporal scale. Tests for the effect of anthropogenic high-temperature heating, similar to that described in Section 4.3, are also being performed, as Site JE0004 had a sequence of hearths (Williams, in preparation). The effect of short-lived heating on these shells is not known at present, but will be the subject of future investigations.

## 5 Conclusions

This study is the first application of the new methodologies of closed system protein geochronology to shell midden deposits. Two main areas were investigated, from contrasting climate zones: Scotland and the Red Sea. Results obtained showed that the technique has the potential to resolve samples of differing age within Holocene deposits in both areas. The tropical temperatures accelerate the rate of protein degradation, precluding direct comparison of the d/l values between samples experiencing very different integrated temperature histories. A possible method for detecting anthropogenic heating of shell samples was also highlighted, potentially helping to detect past exposure to fire.

A relatively simple and quick screening protocol for testing the suitability of molluscan species for AAR dating was described. This test provides a useful tool to inform sampling strategies in the field, demonstrated here by the application to the Red Sea material.

In conclusion, closed system protein geochronology has the potential to be used as a rapid range finder dating technique for shell midden deposits, and is also a reliable and cost-effective alternative to radiocarbon dating for investigating the chronological variability within large clusters of deposits. Where surveys like the Scotland’s First Settlers Project and the Farasan Islands Project have identified large numbers of new sites which need to be dated in order to be interpreted, AAR dating provides a quick reliable method for relative geochronology.

## Figures and Tables

**Table 1:** Provenance of *Patella* samples analysed for AAR dating of shell deposits from Scotland: site name, location, independent age estimate range, number of samples analysed (each identified by a NEaar number), amino acid fraction analysed, Asx and Ser d/l values. Error terms represent one standard deviation around the mean for the site. Each sample was bleached (b), with the free amino acid fraction signified by ‘F’ and the total hydrolysable fraction by ‘H^∗^’.

Site	Location	^14^C age range	NEaar numbers	Fraction	Asx d/l	Ser d/l
Archerfield	Dirleton, East Lothian (66 NT 505 841)	1410–1445 cal AD	5419, 5420, 5421, 5422	bF	0.187 ± 0.012	0.497 ± 0.049
				bH^∗^	0.151 ± 0.004	0.217 ± 0.006
Whitegate Broch	Caithness (ND 3541 6120)	880–1210 cal AD	5423, 5424, 5426	bF	0.270 ± 0.009	0.543 ± 0.062
				bH^∗^	0.176 ± 0.008	0.256 ± 0.006
Coire Sgamhadail 1	Inner Sound, Wester Ross (NG79063826, NG79063826)	2550–1880 cal BC	5505, 5506, 5507, 5508, 5509, 5510	bF	0.374 ± 0.034	0.757 ± 0.047
				bH^∗^	0.210 ± 0.015	0.331 ± 0.032
Sand	Inner Sound, Wester Ross (NG 6841 4934)	7050–6450 cal BC	5493, 5494, 5495, 5496	bF	0.515 ± 0.063	0.840 ± 0.027
				bH^∗^	0.272 ± 0.045	0.411 ± 0.042
Sand (suspected heated)	Inner Sound, Wester Ross (NG 6841 4934)	7050–6450 cal BC	5497, 5498	bF	0.836 ± 0.011	n.d.
				bH^∗^	0.728 ± 0.024	n.d.

**Table 2:** Provenance of *Strombus* samples analysed for AAR dating of shell deposits from the Red Sea: site name, location, specific layer, independent age estimate, number of samples analysed (each identified by a NEaar number), amino acid fraction analysed, Asx, Glx and Ser d/l values. Error terms represent one standard deviation around the mean for the site. Each sample was bleached (b), with the free amino acid fraction signified by ‘F’ and the total hydrolysable fraction by ‘H^∗^’.

Site	Location	^14^C age	NEaar	Fraction	Asx d/l	Glx d/l	Ala d/l
JE0004	Janaba Bay East - Base of the midden	3519–3126 cal BC (BETA-267671; *Chama reflexa*)^a^	5744–5753	bF	0.835 ± 0.030	0.568 ± 0.034	0.810 ± 0.054
				bH^∗^	0.689 ± 0.053	0.446 ± 0.038	0.673 ± 0.056
KM1057	Khur Maadi bay - Base of the midden	3641–3372 cal BC (BETA-255384; *Chama reflexa*)^a^	5754–5763	bF	0.880 ± 0.021	0.586 ± 0.043	0.800 ± 0.038
				bH^∗^	0.744 ± 0.030	0.450 ± 0.032	0.683 ± 0.031
KM1367	Khur Maadi bay - Base of the trench	1520–1270 cal BC (BETA-255386; *Anadara erythraeonensis*)	5262–5265	bF	0.846 ± 0.027	0.481 ± 0.058	0.757 ± 0.064
			5486–5489	bH^∗^	0.640 ± 0.058	0.352 ± 0.031	0.576 ± 0.047

a Radiocarbon dates were obtained on marine shell and adjusted for local marine reservoir effect of 110 ± 38 y (Stuiver and Reimer, 1993).

## Appendix Supplementary information

Supplementary online information accompanies this paper: amino acid composition and racemization data are presented for all the sites considered in this work. The mean and standard deviation for analytical duplicates for each sample are presented (identified by a unique NEaar number). For the heating experiments described in Sections 3.2, 3.3 and 3.4 the raw data are given.
